# Selection and functional identification of a synthetic partial ABA agonist, S7

**DOI:** 10.1038/s41598-019-56343-9

**Published:** 2020-01-08

**Authors:** Myung Ki Min, Rigyeong Kim, Seok-Jun Moon, Yongsang Lee, Seungsu Han, Sangho Lee, Beom-Gi Kim

**Affiliations:** 1 Metabolic Engineering Division, Department of Agricultural Biotechnology, National Institute of Agricultural Sciences, RDA, Jeonju, Jeollabuk-do 54874 Republic of Korea; 2 Department of Biological Sciences, Sungkyunkwan University, Suwon, 16419 Republic of Korea

**Keywords:** Plant sciences, Plant signalling

## Abstract

The stress hormone abscisic acid (ABA) helps plants to survive under abiotic stresses; however, its use as an agrochemical is limited by its chemical instability and expense. Here, we report the development of an *in vivo* screening system to isolate chemicals able to induce ABA signalling responses in rice (*Oryza sativa*) protoplasts. This system consists of an ABA-hypersensitive synthetic promoter containing ABRE and DRE motifs driving a luciferase reporter gene. After efficiently transfecting rice protoplasts with this construct, we screened chemicals library with a similar molecular weight and chemical structure to ABA and identified one chemical, S7, that induced ABA signalling by mediating interactions between the group I and II OsPYL receptors and certain OsPP2CAs in a yeast two-hybrid assay. In an *in vitro* pulldown assay, S7 was found to mediate a weak interaction between OsPYL5/8 and various OsPP2CAs. S7 treatments did not affect seedling growth or seed germination, but could reduce water loss. Rice seedlings treated with S7 exhibited transcriptome profiles that partially overlapped those treated with ABA. Taken together, we concluded that S7 is a new partial ABA agonist, which has potential use in future dissections of ABA signalling and as an agrochemical.

## Introduction

One of the biggest challenges in agriculture is how to improve the productivity of crops under worsening environmental conditions, such as droughts and high temperatures caused by global climate change^1,2^. The plant stress hormone abscisic acid (ABA) and its functional mechanisms have therefore been intensively investigated in an effort to develop new ways of enhancing crop adaptations to stress. ABA is a sesquiterpenoid known to play central roles in adaptive responses to abiotic stresses such as drought, cold, and salt^3,4^. When plants experience abiotic stress conditions, their endogenous ABA levels increase to activate the ABA signalling pathways that regulate adaptive programmes, thereby enabling their survival in the adverse conditions^5^.

The earliest ABA signalling event is triggered by ABA-dependent interactions among three different types of proteins: the ABA receptors named pyrabactin resistance/PYR like/regulatory component (PYR/PYL/RCAR (PYL)) proteins, the clade A phosphatase type 2Cs (PP2CAs), and the subfamily 2 members of SNF1-related protein kinases (SnRK2s). ABA is initially recognized by the ABA receptor PYL. Conformational changes in PYL upon ABA binding enables PYL-ABA to interact with the PP2CAs, thereby form a PYL-ABA-PP2CA complex^6–8^. The formation of this complex releases the SnRK2s from PP2CA-SnRK2 complexes, enabling them to phosphorylate downstream factors such as the ABRE-binding transcription factors or SLOWLY ACTIVATED ANION CHANNEL 1 (SLAC1), which in turn initiate ABA-mediated transcriptional regulation^9–11^.

Although ABA signalling regulates many important abiotic stress response pathways, its potential as an agrochemical to improve stress tolerance in plants is limited by its chemical instability, rapid catabolism, and cost of production^5^. Thus, the development of new ABA analogues or ABA agonists may have important agricultural applications, in addition to their potential use in the research of the ABA signalling pathways^12^. The ABA receptor PYR1 was discovered in chemical genetics studies using pyrabactin, a partial ABA agonist that inhibits seed germination without having any major effect on the vegetative tissues^7,13^. Pyrabactin was the first ABA receptor agonist to be identified; however, since the characterization of the PYL-ABA-PP2CA complex and the ABA-dependent interactions between PYL and PP2CA in yeast assays and *in vitro*, several researchers have identified ABA agonists and antagonists in chemical libraries using a variety of assay systems. Okamoto *et al*. identified the ABA receptor agonist quinabactin in a chemical library using a yeast two-hybrid assay involving a ligand-induced mechanism that controlled the interaction between PYL and PP2CA^14^. Another group independently isolated ABA mimic 1 (AM1), the same molecule as quinabactin, using an AlphaScreen assay technique to screen *in vitro* intermolecular interaction^15^. Nemoto *et al*. advanced the AlphaScreen assay technique and used it in cell-free systems to identify novel ABA receptor agonist julolidine- and fluorine-containing ABA receptor activator 1 (JFA1)^16^. Ito *et al*. discovered RK460, an ABA receptor antagonist, using a chemical assay system^17^, while Zhao *et al*. discovered the ABA antagonist (AA1) using a germination inhibition assay^13^.

In the present study, we use a novel *in vivo* screening system to isolate an ABA receptor agonist from a chemical library. The screening system consists of an ABA-hypersensitive synthetic promoter and a dual-luciferase system using rice (*Oryza sativa*) protoplasts. The isolated chemical mimics the function of ABA to regulate signalling in the guard cells, but did not cause any ABA-related changes to seed germination or seedling growth. We further characterized the chemical to reveal its interaction with a small subset of OsPYLs and OsPP2CAs, through which it induces weak ABA transcriptional responses. We therefore present a new ABA receptor agonist and an efficient screening system by which ABA agonists can be identified *in vivo*. The use of the identified chemical might facilitate the function of select ABA receptors with the PP2C complex, which could be exploited to reduce the water use of crops without impacting yields in non-stress conditions.

## Results

### Development of an ABA-hypersensitive synthetic promoter for use in rice protoplasts

**Figure 1:**
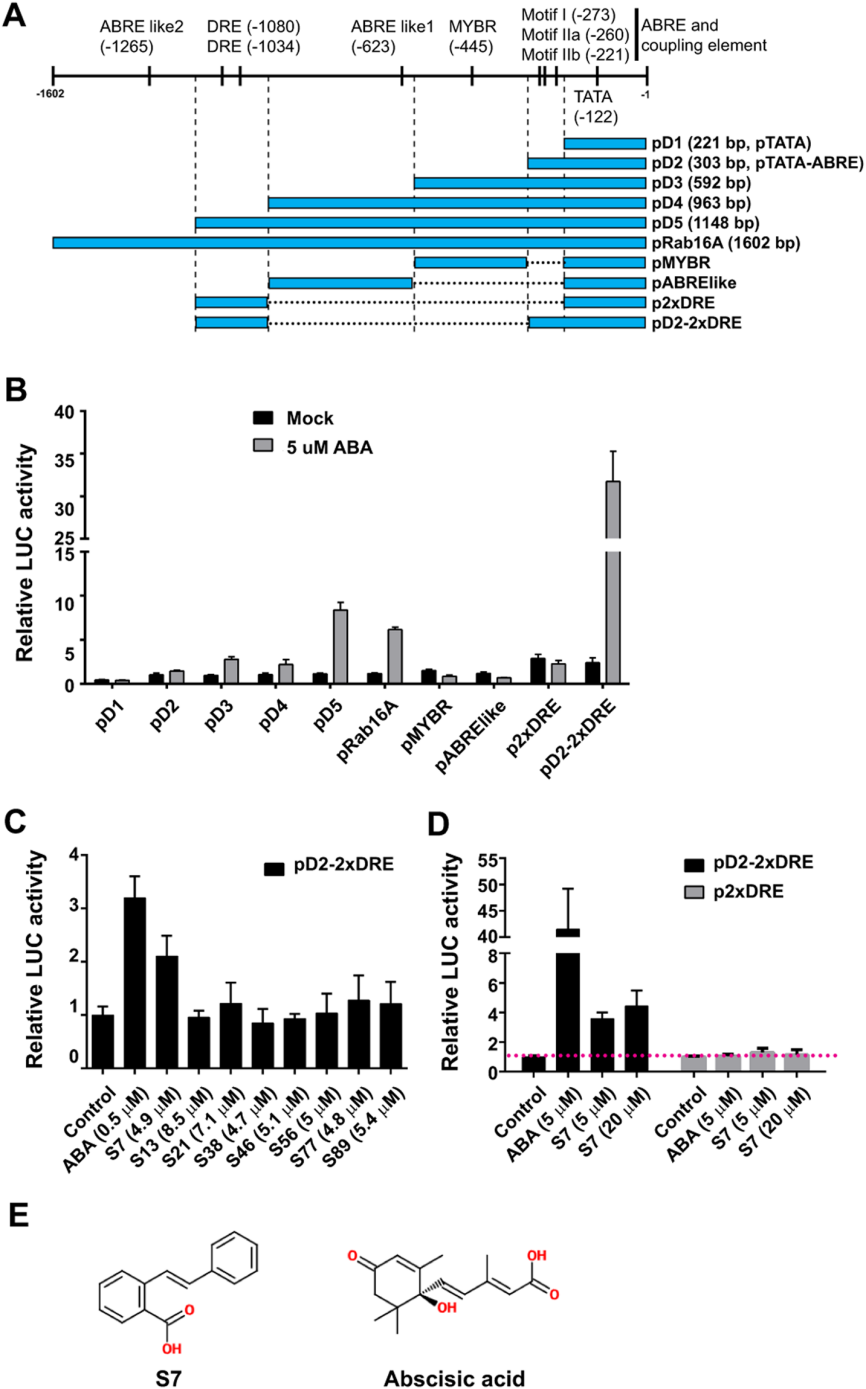
Construction of an ABA-hypersensitive synthetic promoter and its use for the identification of an ABA mimic *in vivo* chemical screen. (**A**) The synthetic promoter constructs and the cis-elements they contain. (**B**) Luciferase (LUC) assay of the ABA sensitivity of several synthetic promoters in rice protoplasts. (**C**) Representative chemical screening results using the *in vivo* screening system for factors regulating ABA signalling in rice protoplasts. (**D**) Comparison of S7 activity in ABA-dependent and ABA-independent signalling, performed using the ABA-sensitive promoter pD2-2 × DRE and the ABA-insensitive promoter pD2, respectively. (**E**) The two-dimensional structures of S7 and ABA. For the LUC assays, each construct harbouring the indicated promoter was introduced into rice protoplasts using the PEG-mediated method. After incubation for 15 h with the indicated chemicals, the LUC activity was detected. The values are the average of three replications and were normalized relative to the control. Error bars indicate ± SD.

*OsRAB16A* promoter-fused luciferase (*OsRAB16A::LUCIFERASE*) was previously used as an ABA-signal-sensing system in rice protoplasts^18^; however, this system may not be sensitive enough for use as a chemical screening system. We therefore screened several different synthetic promoters developed from various elements of the *OsRAB16A* promoter (promoter constructs shown in Fig. 1A), which were fused to the coding sequence of the reporter gene firefly luciferase (*LUC*). All of the promoters possessed a TATA sequence, with other ABA- or abiotic stress-responsive *cis*-acting elements included in a variety of combinations. The various constructs were introduced into rice protoplasts and their promoter activities were assayed. Constructs containing only the TATA sequence (pD1) or a single additional *cis*-acting element, such as a MYB-binding element (pMYBR), an ABA-responsive element (ABRE)-like sequence (pABRE-like), or a dehydration-responsive element (DRE; p2 × DRE), did not respond to the ABA treatments. Unexpectedly, the pD2 promoter containing ABRE and a coupling element (Motif I, Motif IIa, and Motif IIb), which was previously reported to be sufficient for inducing ABA-responsive gene expression, did not respond to ABA in the rice protoplasts^18,19^. The expression of the recombinant pD3 and pD4 promoters, which contained the ABRE-MYBR and ABRE-MYBR-ABRE-like motifs respectively, resulted in a slight increase in luciferase activity following the ABA treatment. The pD5 promoter, which contained an additional DRE *cis*-element in comparison with the pD4 promoter, showed a much stronger response to ABA than pD4. We therefore constructed a promoter combining the two DRE *cis*-elements with the pTATA-ABRE motifs (pD2-2 × DRE). The resulting pD2-2 × DRE promoter had a five-fold increase in luciferase activity following ABA treatment compared with the full *OsRAB16A* promoter (Fig. 1B); therefore, we used this construct (*pD2-2* × *DRE::LUC*) as a reporter of ABA signalling.

### Screening chemicals to induce ABA-mimicking signal responses

The Korea Chemical Bank has 55,000 chemical libraries. We isolated a group of chemicals with a similar structure to ABA by selecting those with a molecular weight lower than 400 Da that possessed a carboxyl group and an aromatic ring structure. A total of 110 such chemicals were identified, and we screened them using our ABA-signal-sensing reporter system. We identified a chemical, SIMILAR TO ABA 7 (S7), which could activate the pD2-2 × DRE promoter, albeit to a much lower level than ABA (Fig. 1C,D). To identify whether S7 activate ABA independent or ABA dependent signal, we compared the responses of two reporters to S7, the ABA independent signal reporter (p2 × DRE which contains only TATA motif and 2xDRE cis-elements) and the ABA signal reporter, pD2-2xDRE. The S7 treatment activated pD2-2 × DRE, but did not activate the p2xDRE promoter (Fig. 1D). This result suggests that S7 activates an ABA-dependent signal transduction pathway.

### Physiological effects of S7 in rice

**Figure 2:**
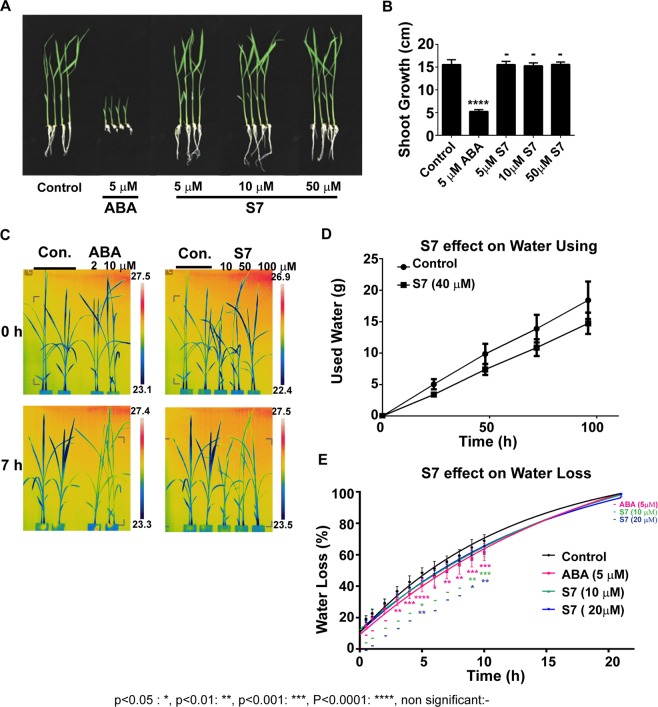
Physiological effects of S7 in rice. (**A**,**B**) Seedling growth assay using S7 or ABA treatments. For (**B**), n = 10, error bars indicate ± SD. Three independent replicates were performed. (**C**) Leaf temperatures of 4th-leaf-stage plants measured using infrared. (**D**) Water use by hydroponically grown plants. The water consumed by two plants per test tube was measured. n = 6, error bars are ± SD from three biological repeats. (**E**) Gravimetric assay of water lost from detached leaves. The curves were fitted with an exponential decay. The treated plants were compared with the control at each time point using a two-way ANOVA. n = 5, error bars are ± SD.

To demonstrate whether S7 has similar physiological functions to ABA, we investigated the growth of young seedlings in addition to performing a water loss assay. Unexpectedly, S7 did not show any effect on young seedling growth even when high concentrations of the chemical were used (up to 50 μM; Fig. 2A,B). In contrast, ABA severely restricted seedling growth even at relatively low concentrations (5 μM). We measured the rate of water loss in detached leaves after treatment with S7 or ABA, revealing that S7 caused similar rates of water loss to ABA at some time points(5, 9 and 10 h) even though S7 has lower activities than ABA (Fig. 2E). For further confirmation of this effect, we observed the temperatures of leaves treated with these two compounds using an infrared camera. In plants treated with variable concentrations of either ABA or S7, the temperatures of the leaf blades increased by more than 1 °C after 7 h (Fig. 2C), suggesting that, like ABA, S7 may cause stomatal closure. We also measured the water use of plants treated with S7, and found they consumed an average of 25% less water in comparison with the control (Fig. 2D). We also measured stomata apertures using Arabidopsis to get the direct evidence that S7 induces stomata closing. Expectedly S7 induce the stomata closing strongly (Supplementary Fig. 2). In addition, we also measured the stomata conductance of *Brassica rapa* after treatment of ABA, S7 or pyrabactin. That result was confirmed by a detection of infrared image and measuring of stomatal conductance (Supplementary Fig. 3). These experiments revealed that S7 did suppressed water consumption and water loss by enhancing the stomatal closure at both dicot and monocot plants in a similar manner to ABA even though the activity is lower than ABA.

### S7 mediates the interaction between a subset of OsPYLs and OsPP2CAs

**Figure 3:**
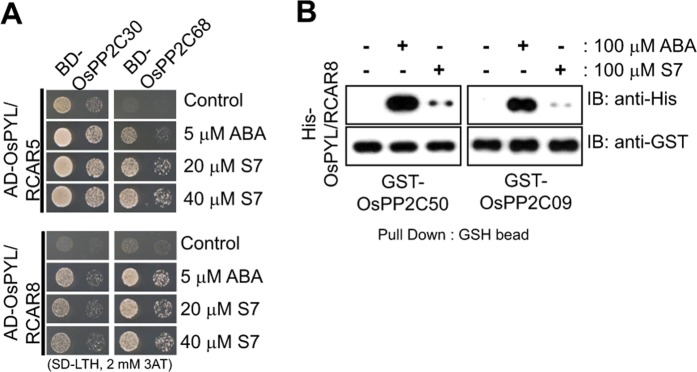
S7 mediated interaction between OsPYLs and OsPP2CAs. (A) Yeast two-hybrid assay. The droplets on the left were produced using a 1:10 dilution of yeast grown to saturation in SD (−) LT, while those on the right were diluted 1:100. (**B**) Pull-down assay of GST-tagged proteins using GSH-conjugated agarose beads. The proteins were detected using a western blot analysis with anti-His mouse antibodies and anti-GST rabbit antibodies. The full length blot is presented in Supplementary Fig. 4.

ABA mediates the physical interaction between the OsPYLs and the OsPP2CAs to initiate its signalling pathway^6,7^. We therefore tested whether S7 induces direct interactions between 10 OsPYL/RCARs and 9 OsPP2CAs using yeast two-hybrid assays. Many combinations showed ABA-independent interactions (Supplementary Fig. 1), as was previously reported in *Arabidopsis thaliana*. Excluding these non-ABA-related interactions, we found that some combinations of OsPYL/RCARs and OsPP2CAs underwent ABA- or S7-dependent interactions. The ABA receptors were found to interact with OsPP2C30 or OsPP2C68 in yeast grown on plates containing ABA or S7 (Fig. 3A).

We also confirmed the interactions between the OsPYL/RCARs and the OsPP2Cs using an *in vitro* pulldown assay. In the presence of ABA or S7, GST-OsPP2C50 or GST-OsPP2C09 were found to directly bind to His-OsPYL/RCAR8, although their affinity was lower in the presence of S7 than in the presence of ABA (Fig. 3B and Supplementary Fig. 4). The results showed that S7 directly mediates the interaction between some OsPYL/RCARs and OsPP2CAs, although it was not able to induce every ABA-mediated interaction between these proteins.

### Comparison of gene expression profiles in ABA- and S7-treated plants

**Figure 4:**
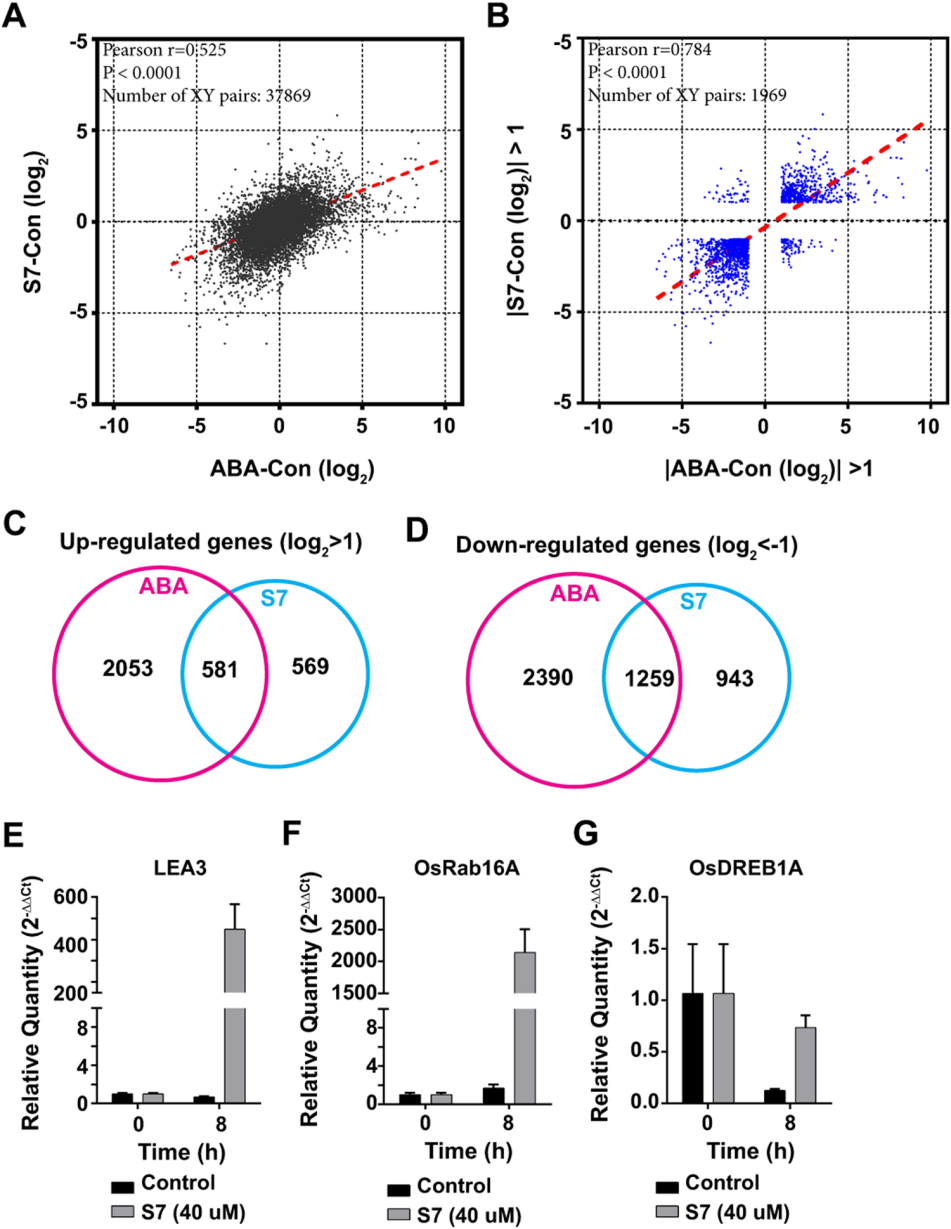
Comparison of gene expression profiles in ABA- and S7-treated plants. (**A**,**B**) Correlation analysis of the transcriptomes of whole plants treated with S7 or ABA for 7 h. the axes are the differential expressions under ABA (x axis) or S7 (y axis) treatments compared with the control. (**A**) Correlation analysis for whole transcripts. (**B**) Correlation analysis for transcripts showing a two-fold change or greater in plants treated with S7 or ABA. Blue dots indicate the genes showing a log_2_ fold change >1 and <−1. All reads were transformed to log_2_ after normalized by DESeq2 program. The Pearson correlation coefficients (r) for each comparison are shown on the graphs. (**C**,**D**) Venn diagrams representing genes up-regulated (log_2_ fold change >1) or down-regulated (log_2_ fold change <−1) by ABA or S7. (**E**–**G**) The expression levels of *LEA3*, *OsRAB16A*, and *OsDREB1A* after a treatment with S7. The transcription levels were analysed using RT-qPCR with gene-specific primers, and were normalized to the expression level of *UBIQUITIN5*. The transcript values are represented relative to the levels in the control. Error bars are ± SD (n = 3).

To compare the ABA- and S7-mediated transcriptional regulation of gene expression, we conducted a transcriptomic analysis of plants treated with ABA or S7 for 7 h using RNA-sequencing. All reads of transcripts were normalized and then transformed to log_2_. The values of differences were determined by difference between ABA or S7 treated transcripts and the non-treated transcripts (control; ABA-con and S7-con). Our analysis of the correlations between these transcriptomes revealed a moderate positive relationship (*r* = 0.525; Fig. 4A). When we evaluated only genes with more than two-fold difference in expression (|log_2_| > 1) between plants subjected to the different treatments, the correlation was much stronger (*r* = 0.784; Fig. 4B). The slope of this relationship indicated that S7 induced lower levels of gene expression than ABA. A total of 2639 genes and 1150 genes were found to be upregulated by the treatments with ABA and S7, respectively, with more than half of the S7-induced genes (581 genes) also induced by ABA (Fig. 4C). Conversely, 3649 genes and 2202 genes were found to be downregulated by ABA and S7, respectively, with more than half of the S7-repressed genes (1259 genes) found to be commonly repressed by ABA (Fig. 4D). The upregulated and downregulated genes are listed in Supplementary Table 2.

To validate the results of the transcriptomic analysis, we conducted an RT-qPCR analysis of representative ABA-inducible and ABA-independent genes using the total RNA of plants treated with S7 for 7 h. The expression levels of the three marker genes (Os*LEA3*, ABA inducible; *OsRAB16A*, inducible by both ABA signalling and ABA-independent abiotic stresses; and *OsDREB1A*, ABA-independent cold-inducible) were evaluated using gene-specific primers (Supplementary Table 1). As expected, *LEA3* and *OsRAB16A* were induced to significantly higher levels (more than 100-fold higher) in the S7-treated plants than in the control; however, *OsDREB1A* expression was not induced by S7, although its expression level was higher than in the control (Fig. 4E–G).

### S7 can activate ABA signalling mediated by the ABA receptors and PP2CAs

As shown above, S7 was able to mediate some of the interactions between the ABA receptors and the PP2CAs, and may also regulate the expression of some ABA-responsive genes. We therefore investigated whether S7 activates ABA signalling via the interactions between the ABA receptors and PP2CAs, using our ABA-signal-sensing system in rice protoplasts to transiently overexpress ABA signalling components.

**Figure 5:**
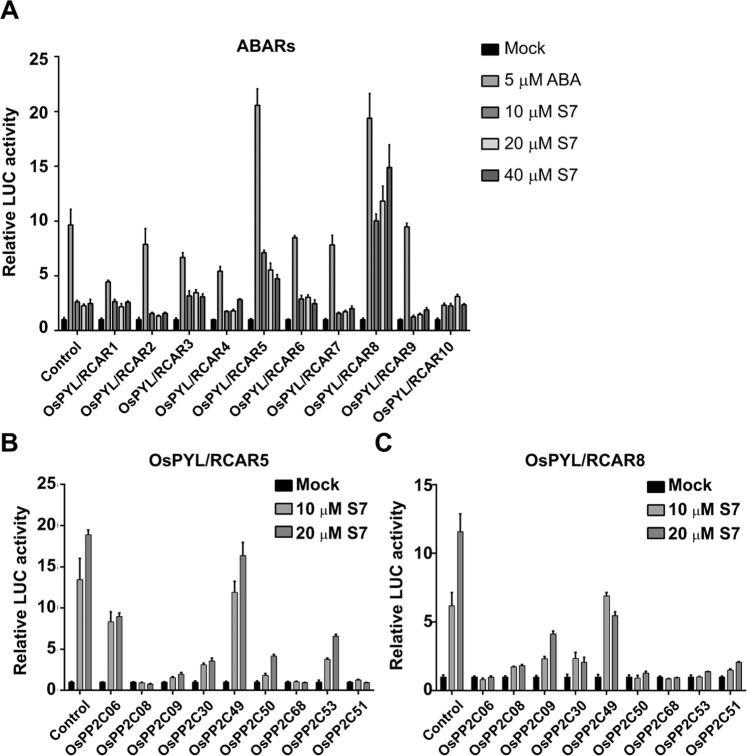
S7 affects ABA signalling mediated by ABA receptors and PP2CAs in rice. (**A**) Screening of ABA receptors activated by S7. (**B**) Screening of OsPP2CAs paired with OsPYL/RCAR5 in signalling mediated by S7. (**C**) Screening of OsPP2CAs paired with OsPYL/RCAR8 in signalling mediated by S7. The values represent the values relative to those of the control plants. Error bars are ± SD (n = 3).

We first transiently overexpressed nine ABA receptors together with the reporter plasmid (*pD2-2* × *DRE::LUC*) in rice protoplasts, which were treated with ABA or various concentrations of S7. The ABA treatments induced the LUC activity in all protoplasts (including the control), reaching levels 8- to 20- fold higher than the control except in the protoplasts overexpressing OsPYL/RCAR10 (Fig. 5A). S7 also induced increases in LUC activity in all lines relative to the control; however, these differences were much less dramatic than for ABA. The largest increases in LUC activity in the S7-treated protoplasts were observed in the lines overexpressing *OsPYL/RCAR8*, which showed a 10- to 15-fold increase, and the line overexpressing *OsPYL/RCAR5*, which showed a 4.7- to 7.1-fold increase. OsPYL/RCAR5 and OsPYL/RCAR8 were therefore selected as candidates for the major target of S7.

We then co-expressed each of the nine PP2CAs together with the genes encoding the ABA receptors OsPYL/RCAR5 (Fig. 5B) or OsPYL/RCAR8 (Fig. 5C). S7 significantly induced the LUC activity in protoplasts expressing *OsPYL/RCAR5* alongside *OsPP2C06*, *OsPP2C53*, *OsPP2C50*, or *OsPP2C49*, and induced LUC activity in protoplasts expressing *OsPYL/RCAR8* with either *OsPP2C09* or *OsPP2C49*. These results mean that OsPYL/RCAR5 and OsPYL/RACAR8 interact with different OsPP2CA partners in response to S7 treatment, and indicates that S7 can partially activate ABA signalling by inducing the interactions of specific OsPYL/RCARs and OsPP2CAs.

## Discussion

Following the characterization of the ABA receptor-ABA-PP2C complex structures, ABA agonists and antagonists were rapidly identified using structure-based chemical design approaches and several screening systems based on the characteristics of the interactions between the ABA receptors and the PP2CAs^16,17,20–24^. Here, we developed a highly sensitive screening method to directly screen the *in vivo* activity of chemicals in rice protoplasts.

Previous studies have shown that, in the *OsRAB16A* promoter, the presence of repeated ABRE and CE1 motifs is necessary and sufficient for inducing the expression of its associated gene in response to ABA in rice^19,25,26^. Despite this, our analysis of recombinant promoters containing various combinations of ABA-responsive elements suggests that ABRE and CE1 either cannot induce ABA responsiveness in rice protoplasts, or they induce it to a very low level. Instead, we found that both ABREs and DREs were required to fully induce the ABA-responsive genes in rice protoplasts. DREs are known to function as a coupling element for the ABREs in Arabidopsis, and we found that the removal of several other elements between the ABREs and DREs increased the ABA responsiveness of the recombinant promoters^27^. We used the synthetic promoter pD2-2 × DRE to develop a highly sensitive screening system for monitoring ABA responses. This enabled us to identify S7 as a partial ABA agonist in signalling and interactions between the ABA receptor and PP2CAs, despite its low activity relative to ABA.

S7 cannot fully activate all ABA receptors and it was therefore hypothesized that S7 mediates the interactions between specific group III ABA receptors and a selection of PP2CAs^6^. Even using high concentrations of S7, we could not clearly determine all the targets of this compound; therefore, it might mediate very weak interactions and have minor signalling effects. S7 treatments did not have any physiological effects on seed germination and young seedling growth; however, it had affected stomatal closure and transpiration not only in monocot plants but also in dicot plant. Conversely, a previously reported ABA agonist, pyrabactin, also functions as a partial ABA agonist for a subset of ABA receptors, but only produces effects on seed germination, not on stomatal regulation (Supplementary Fig. 3)^7^. Quinabactin, another ABA agonist, preferentially targets the group I ABA receptors and can mimic ABA in all of its physiological effects, including inhibiting seed germination and seedling growth, and promoting stomatal closure^14,15^. Taken together with the observed effects of other ABA agonists, our results suggest that S7 may target the group III ABA receptors and some PP2Cs, which might mainly function in stomatal regulation. Another suggestion is that such weak signalling is not sufficient to invoke the dramatic physiological effects of the ABA-mediated inhibition of seed germination and growth inhibition, but that stomatal signalling might be more sensitive to ABA than these other physiological effects and can therefore be influenced by S7.

Around half of the genes up- or down-regulated by the S7 treatments were also differently expressed after treatments with ABA. Although S7 regulated the expression of many ABA-responsive genes, these transcriptional changes were induced to a much lesser extent by S7 than by ABA, which provides further evidence that S7 is a partial and weak mimic of ABA. The transcriptional changes induced by the ABA agonist quinabactin were reported to be quite similar to those induced by ABA, resulting in similar transcriptome profiles; however, the very weak partial ABA agonist pyrabactin induced similar transcriptomic changes to ABA in the seed germination stages but not in the seedlings^7,14,15^. The correlation r value between ABA and pyrabactin was 0.72 in the seedlings, which was lower than the correlation value we calculated for S7- and ABA-induced transcriptome changes in the present study^7^. These findings suggest that S7 is a partial ABA agonist, with a functional similarity to ABA that lies somewhere between that of pyrabactin and quinabactin.

Taken together, we found out the new partial ABA agonist which might function in stomatal regulation. This chemical has a potential to be used as an agrochemical and a tool to dissect ABA signalling.

## Methods

### Plant growth conditions and chemical library

The rice cultivar Dongjin (*Oryza sativa ssp*. *japonica* cv. Dongjin) was used in this study. Husked rice seeds were sterilized with 70% ethanol (30 s), then with 2% NaClO (40 min), and finally were washed five times with distilled water. The sterilized seeds were grown on a 1/2-strength MS (Murashige and Skoog; Duchefa, Netherland) medium containing 0.4% phytagel (adjusted to pH 5.8 with 0.5 g/L MES and KOH) in long-day conditions (16 h light and 8 h dark) at 28 °C.

The seeds of *Brassica rapa* and Arabidopsis were sterilized with washing of 70% Ethanol (30 s) and 2% of NaClO (30 min). The Brassicas were grown on a 1/2-strength MS (Murashige and Skoog; Duchefa, Netherland) medium containing 0.4% phytagel (adjusted to pH 5.8 with 0.5 g/L MES and KOH) in long-day conditions (16 h light and 8 h dark) at 23 °C. The chemicals used in the screen were obtained from the Korea Chemical Bank, and were dissolved in DMSO to make stock solutions.

### Physiological assays

For the rice seedling growth assay, 3-day-old seedlings grown in 1/2 MS were transferred into 1/2 MS containing 5 μM ABA or various concentrations of S7, then incubated in long-day conditions at 28 °C for 7 days before observation.

For the rice leaf temperature measurements, non-husked seeds were sterilized with 0.01% procholraz and 0.01% fludioxonil for 24 h, washed with distilled water, then germinated on wet filter paper. Five-day-old plants were transferred to a culture box containing Yosida solution^28^ in long-day conditions at 28 °C. The leaf temperatures of 12-day-old rice seedlings (4^th^ leaf stages) treated with various concentrations of ABA or S7 were measured with an infrared camera (FLIR P620; FLIR Systems, USA) at the indicated times.

To measure the amount of water used by rice treated with S7 or ABA, 12-day-old plants grown in Yosida solution in long-day conditions were transferred to closed tubes containing 40 ml tap water, without or with various concentrations of the compounds of interest. The total weight of each tube was measured at the time intervals indicated. This experiment was conducted in triplicate for more than six individuals.

For the water loss assay of rice, 12-day-old plants were treated with ABA or S7 for 7 h, after which the fresh weights of the detached leaf blades (third leaf) were measured over the course of a day.

For obtaining of infrared brassica image and measurement of stomatal conductance, *Brassica rapa* were transferred to water from MS media and then were adapted to the outside environment for 6 days. After chemical treatment, the plants were incubated at 28 °C for 4 h in light condition. The infrared images were obtained by FLIR p620 (FLIR^(R)^ systems). The stomatal conductance was obtained by LI-6400XT (LI-COR Biosciences). The detections were performed for 5 min at each sample under condition which is 550 μmol mol^−1^ of CO_2_, 50–60% relative humidity and natural light condition.

### Vector constructions

The previously reported ABA-inducible *OsRAB16A* (*Os11g26790*) promoter was reorganized based on the cis-acting element motifs to assay the ABA responsiveness of these fragments both alone and in combination. For the generation of the promoter constructs, specific sequences were amplified using PCR (gene-specific primers listed in Supplementary Table 1). The amplified PCR products were substituted into the promoter region of *pRC29A-LUC-NOS* using the *Bam*HI and *Nco*I restriction enzyme sites.

The effector plasmids encoding various rice ABA receptors were constructed by amplifying the genes encoding these receptors using PCR (gene-specific primers listed in Supplementary Table 1) and inserting them into the pENTR-D-TOPO plasmid (Thermo Fisher Scientific, USA). The insertions were then transferred into the pGEM-gw-Flag using LR recombination (Thermo Fisher Scientific, USA). pGEM-gw-Flag was constructed by cloning a maize *UBIQUITIN* promoter expression cassette into a pGEM-T easy vector, alongside a gateway cloning cassette amplified from a pGA2897 vector using PCR with the Ubi5-F and Flag-R primers (Supplementary Table 1).

### Luciferase assay using rice protoplasts

The rice protoplasts were isolated using the protocol described by Kim *et al*.^18^. For the luciferase assay, the firefly luciferase (fLUC) and renilla luciferase (rLUC) plasmids and effector plasmids were introduced into the purified rice protoplasts as previously described^18^. A total of 5 μg ABA-responsive fLUC reporter plasmid, 0.5 μg constitutively expressed rLUC reporter plasmid (control), and 5 μg of each effector plasmid were transfected into the protoplasts. After 15 h incubation, the expressed luciferases were detected using a dual luciferase assay kit (Promega, USA) and a GloMax 96 Microplate Luminometer (Promega, USA), according to the manufacturer’s instructions.

### Reverse-transcription quantitative PCR (RT-qPCR)

For the RT-qPCR analysis, first-strand cDNA was synthesized from 4 μg of total RNA using SuperScript III reverse transcriptase (Thermo Fisher Scientific, USA). A 1:40 dilution of total cDNA was used for the RT-qPCR. The amplification parameters were as follows: 15 min of denaturation and enzyme activation at 95 °C; followed by 40 cycles of 95 °C for 5 s, 60 °C for 15 s, and 72 °C for 30 s; with a final step performed at 65–95 °C (1 °C/s) for the melting curve analysis. The amplified signals were detected using a MyiQ real-time PCR system (Bio-Rad Laboratories, USA) using SYBR Premix Ex Taq^TM^ (TOPrealTM qPCR 2X PreMix, enzynomics, Korea). The data were normalized based on the expression of rice *UBIQUITIN5*, and the relative gene expression was analysed using the 2^−ΔΔCT^ method. At least three biological repetitions were performed. The primer sequences used for RT-qPCR analysis are listed in Supplementary Table 1.

### Yeast two-hybrid assay

Yeast two-hybridization assays were performed using the Matchmaker^TM^ GAL4 Two-Hybrid System 3 (Clontech, USA), according to the manufacturer’s manual. The lithium acetate method was used to introduce pGADT7 (encoding the activation domain) and pGBKT7 (encoding the binding domain) plasmids into yeast (*Saccharomyces cerevisiae* strain AH109). The yeast were grown on the Yeast Minimal Media/Synthetic Defined (SD) media (Clontech, USA) without leucine and tryptophan, then transferred to selection media lacking leucine, tryptophan, and histidine but supplemented with 2 mM 3-amino-1,2,4-triazole (3-AT) (Sigma-Aldrich, USA). For the yeast spot assays, exponentially grown yeast cells were harvested and adjusted to OD_600_ = 0.5 using sterilized water, and then further diluted (1:10 and 1:100). Yeast cells were spotted onto the SD medium without leucine and tryptophan, and the SD medium without leucine, tryptophan, or histidine. Their growth was observed after three days.

### Transcriptomic analysis

For the RNA-sequencing (RNA-seq) analysis, 14-day-old plants grown on 1/2 MS media were transferred into tap water containing 10 μM ABA or 30 μM S7. After 7 h incubation, total RNA was extracted from the shoot and purified using the RNeasy Mini Kit (Qiagen, Germanyt). The quality and quantity of the extracted RNA was checked using an Agilent Technologies 2100 Bioanalyzer (Agilent Technologies, USA); the RNA integrity number values of all samples were greater than or equal to 7. The libraries for sequencing were prepared using a TruSeqRNA Sample Prep Kit v2 (Illumina, USA), following the manufacturer’s instructions. The sequencing of the libraries was performed using a HiSeq. 4000 system (Illumina, USA) generating single-end 101-bp reads. The trimmed reads were mapped to IRGSP (v1.0) and assembled into transcripts. The expression values were determined using the StringTig program, and then normalized by DESeq2 program^29^. The graphs were constructed using GraphPad Prism6 program.

### Recombinant protein expression and GST pull-down assay

Rosetta 2 (DE3) (Novagen) containing plasmids encoding proteins used in this study was cultured in LB media and overexpressed with 0.1 mM IPTG for 12 h at 16 °C with gentle shaking. Harvested cells were resuspended to lysis buffer (20 mM Tris-HCl pH 8.0, 200 mM NaCl, 0.1 mM tris(2-carboxyethyl)phosphine (TCEP), 0.05% (v/v) β-mercaptoethanol and 0.05% (v/v) Triton X-100) and lysed by ultrasonication. GST and 6xHis tagged proteins were purified by affinity chromatography using glutathione-Sepharose 4B resin (GE HealthCare) and Ni-NTA resin (Qiagen), respectively. Purified proteins were dialyzed to final storage buffer (20 mM Tris-HCl pH 8.0, 200 mM NaCl, 0.1 mM TCEP, 10% (v/v) glycerol). 1 mM MgCl_2_ was supplemented to the final storage buffer in case of GST-PP2C09 and 50 proteins. Purified GST-PP2C09 and 50 proteins were used as baits for GST pull-down assay. Firstly, 2 μg of GST-PP2C proteins were added to pull-down buffer (20 mM Tris-HCl pH 8.0, 1 mM MgCl_2_, 0.1 mM TCEP, 0.1 mg/ml BSA and 1 mM phenylmethylsulfonyl fluoride) with pre-equilibrated glutathione-Sepharose 4B resin (GE HealthCare). 100 μM of ABA or S7 were supplemented to pull-down buffer to investigate ligand-mediated protein-protein interaction. We incubated at room temperature for 1 hour with gentle agitation. Next, 7 μg of His-OsPYL/RCAR8 was added as a prey protein with additional incubation at 4 °C for 2 hours. Incubated resins were washed by pull-down buffer, with ABA and S7 if needed. Pulled-down proteins were eluted from resins by pull-down buffer with 10 mM reduced glutathione. SDS-PAGE and western blot with anti-His and anti-GST antibodies (Santa Cruz) were performed.

## Supplementary information

supplementary table and Figures

Supplementary Table 2
