# Supplementary material for: Selection and functional identification of a synthetic partial ABA agonist, S7
Source: Sci Rep. 2020 Jan 8;10:4. doi: 10.1038/s41598-019-56343-9 (PMC6949257; doi:10.1038/s41598-019-56343-9)
Supplement: Supplementary file 1 — supplementary table and Figures [file 41598_2019_56343_MOESM1_ESM.docx]

**Selection and functional identification of a synthetic partial ABA agonist, S7**

**Myung Ki Min^1^, Rigyeong Kim^1^, Suk-Jun Moon^1^, Yongsang Lee^1^, Seungsu Han^2^, Sangho Lee^2^, and Beom-Gi Kim^1*^**

| **Supplementary Table 1. List of primers for construction and RT-qPCR analysis.** | | |
| --- | --- | --- |
|  |  |  |
| **Name** | **Forward-primer** | **Sequence** |
|  | **Reverse-primer** |  |
| **pD1** | pD1-F | GGATCCGCCACCGTCCACCGGCTCC |
|  | pRab16A-R | CCATGGCCTGCTTAAGCTAAAGCTGA |
| **pD2** | pD2-F | GGATCCTGTCTTCGAGAAACGCCTC |
|  | pRab16A-R | CCATGGCCTGCTTAAGCTAAAGCTGA |
| **pD3** | pD3-F | GGATCCCTGACCACCAGTTGAAAGGT |
|  | pRab16A-R | CCATGGCCTGCTTAAGCTAAAGCTGA |
| **pD4** | pD4-F | GGATCCCAGCTTGCTTATCTCTCCCAT |
|  | pRab16A-R | CCATGGCCTGCTTAAGCTAAAGCTGA |
| **pD5** | pD5-F | GGATCCGACCACGCTAGTGACCATGA |
|  | pRab16A-R | CCATGGCCTGCTTAAGCTAAAGCTGA |
| **pRab16A** | pRab16A-F | GGATCCCTGTAGAGAGGATGACCCTTGTCACC |
|  | pRab16A-R | CCATGGCCTGCTTAAGCTAAAGCTGA |
| **pMYBR** | pMYB-TATA-F | (ATGAGTCACCACCCG)GCCACCGTCCACCGGCTCC |
|  | fLUC312-R | GGGCGCAACTGCAACTCCGA |
|  | pD3-F | GGATCCCTGACCACCAGTTGAAAGGT |
|  | pTATA-MYB-R | (CCGGTGGACGGTGGC)CGGGTGGTGACTCATCGGG |
|  | pD3-F | GGATCCCTGACCACCAGTTGAAAGGT |
|  | pRab16A-R | CCATGGCCTGCTTAAGCTAAAGCTGAA |
| **pABARlike** | pABRElike-TATA-F | (AGTCCCCAAGTGAAA)GCCACCGTCCACCGGCTCC |
|  | fLUC312-R | GGGCGCAACTGCAACTCCGA |
|  | pD4-F | GGATCCCAGCTTGCTTATCTCTCCCAT |
|  | pTATA-ABRE-R | (CCGGTGGACGGTGGC)TTTCACTTGGGGACTGCTATG |
|  | pD4-F | GGATCCCAGCTTGCTTATCTCTCCCAT |
|  | pRab16A-R | CCATGGCCTGCTTAAGCTAAAGCTGAA |
| **p2xDRE** | pTATA-2xDRE-F | (CCTCATGTACACAAT)GCCACCGTCCACCGGCTCC |
|  | fLUC312-R | GGGCGCAACTGCAACTCCGA |
|  | pD5-F | GGATCCGACCACGCTAGTGACCATGA |
|  | pTATA-2xDRE-R | (CCGGTGGACGGTGGC)ATTGTGTACATGAGGACAAGGG |
|  | pD5-F | GGATCCGACCACGCTAGTGACCATGA |
|  | pRab16A-R | CCATGGCCTGCTTAAGCTAAAGCTGAA |
| **pD2-2xDRE** | p2xDRE-D2-F | (CCTCATGTACACAAT)TGTCTTCGAGAAACGCCTCG |
|  | fLUC312-R | GGGCGCAACTGCAACTCCGA |
|  | pD5-F | GGATCCGACCACGCTAGTGACCATGA |
|  | pD2-2xDRE-R | (CGTTTCTCGAAGACA)ATTGTGTACATGAGGACAAGGG |
|  | pD5-F | GGATCCGACCACGCTAGTGACCATGA |
|  | pRab16A-R | CCATGGCCTGCTTAAGCTAAAGCTGAA |
| **pGEM-gw-Flag** | Ubi5-F | CTG CAG TGC AGC GTG ACC |
|  | Flag-R | CGGGTACCTTACTTGTCATCGTCGTCCTTGTAGTCTCCTCCGGCGATATCGACCACTTTGT |
| **OsPYL/RCAR3** | RCAR3-F | CACCATGGTGGAGGTGGGAGGAGG |
|  | RCAR3-R | CCGGTCGAGGGGCTCGGT |
| **OsPYL/RCAR5** | RCAR5-F | CACCATGGTGGGGCTTGTGGGAGG |
|  | RCAR5-R | CTGTTCAAGTGGCGAGGT |
| **OsPYL/RCAR4** | RCAR4-F | CACCATGCCGTGCATCCCGGCGTC |
|  | RCAR4-R | CGAGCCGGCGGCCCTCG |
| **OsPYL/RCAR9** | RCAR9-F | CACCATGGAGGCGCACGTGGAGAG |
|  | RCAR9-R | GTCGCGCCGCCGCGAAGC |
| **OsPYL/RCAR10** | RCAR10-F | CACCATGGAGCAGCAGGAGGAAGT |
|  | RCAR10-R | TTTCGGCTGCCGCCGG |
| **OsPYL/RCAR2** | RCAR2-F | CACCATGGAACCACACATGGAACG |
|  | RCAR2-R | TGGTTACCCGCCGCTGGCGG |
| **OsPYL/RCAR1** | RCAR1-F | CACCATGCCGTACGCCGCCGTACG |
|  | RCAR1-R | TGCATGATCGATCGATCCGT |
| **OsPYL/RCAR6** | RCAR6-F | CACCATGATGCCGTACACCGCTCC |
|  | RCAR-R | GGCGGCGCGCGGCGCGGCGA |
| **OsPYL/RCAR7** | RCAR7-F | CACCATGAACGGCGCTGGTGGTGC |
|  | RCAR7-R | AGGATTGGCAAGGCGCTCCT |
| **OsPYL/RCAR8** | RCAR8-F | CACCATGAACGGCGTTGGTGGGGC |
|  | RCAR8-R | AGGATTGGCAAGGCGCTCCT |
| **LEA3 (qPCR)** | qLEA3-F | GCCGTGAATGATTTCCCTTTG |
|  | qLEA3-R | CACACCCGTCAGAAATGCTCC |
| **OsRab16A (qPCR)** | qRab16A-F | GAGTACGGCAACCCGGTC |
|  | qRab16A-R | GTCTTGTGCTCCTCCCTCAT |
| **OsDREB1A (qPCR)** | qDREB1A-F | GACGTCCTGAGTGACATGGG |
|  | qDREB1A-R | AGTAGCTCCAGAGTGGGACG |


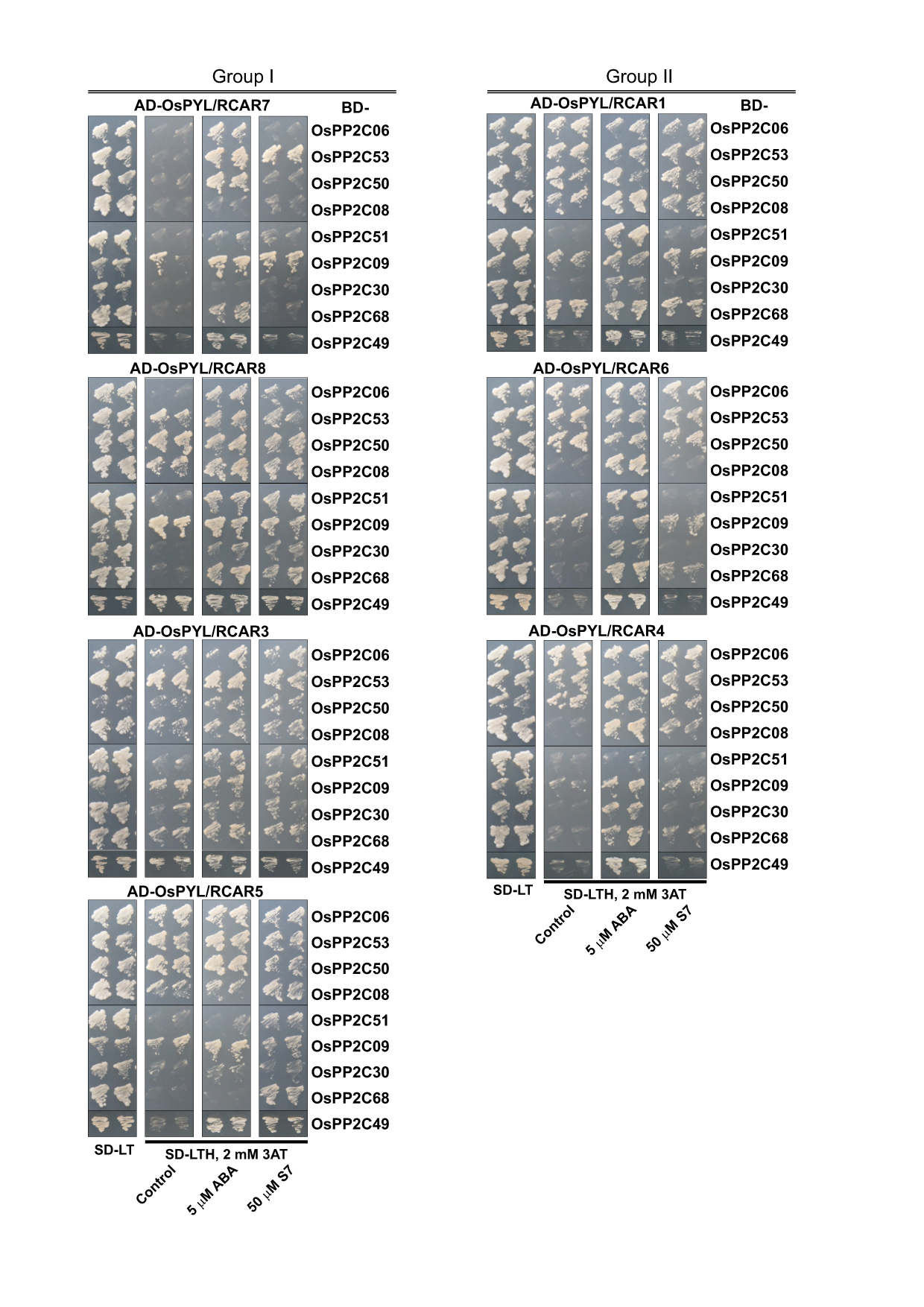


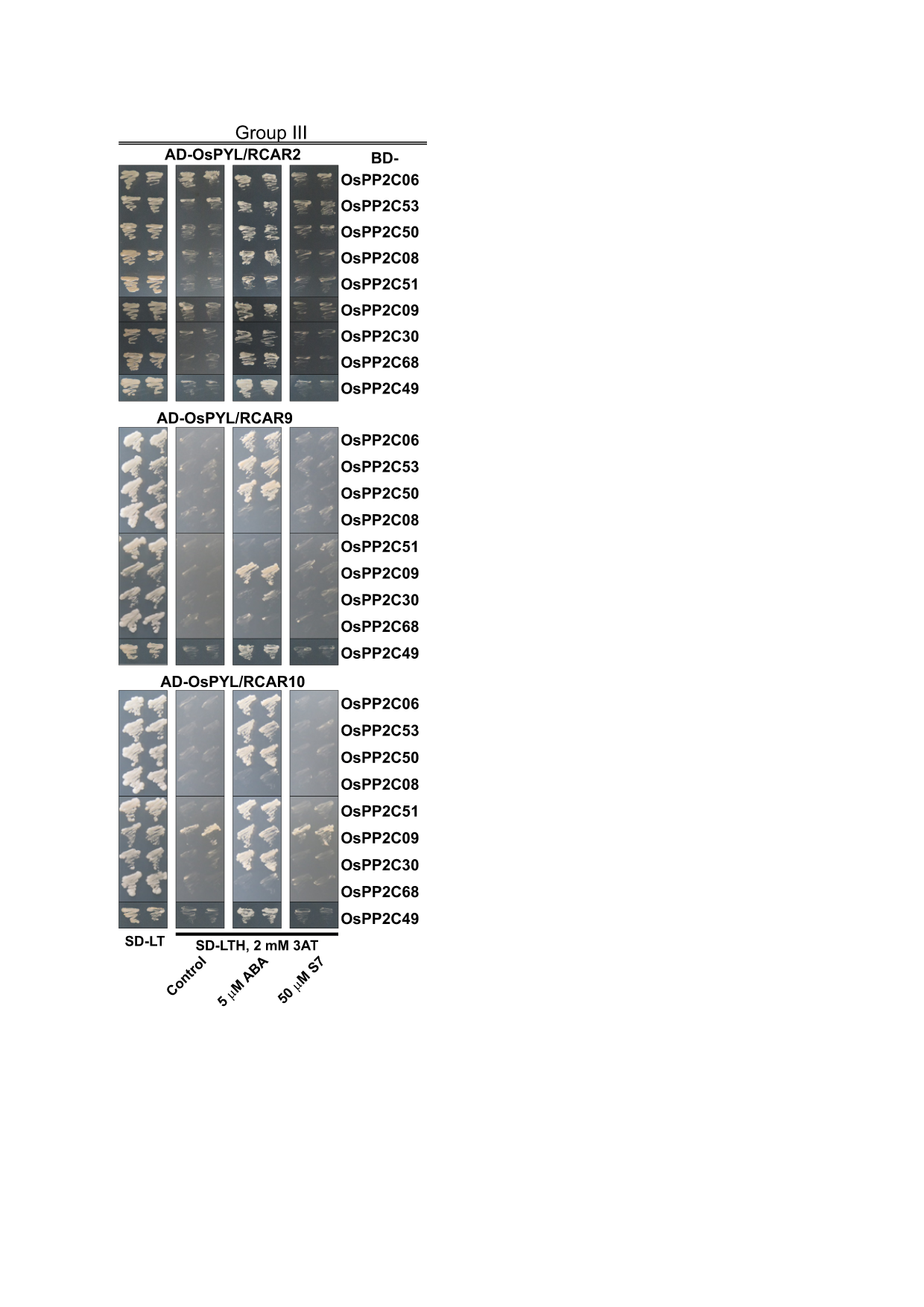


**Supplementary Figure 1. Interaction test of rice Clade A group of OsPP2Cs and 10 ABA receptors in Yeast two-hybrid assay**

**
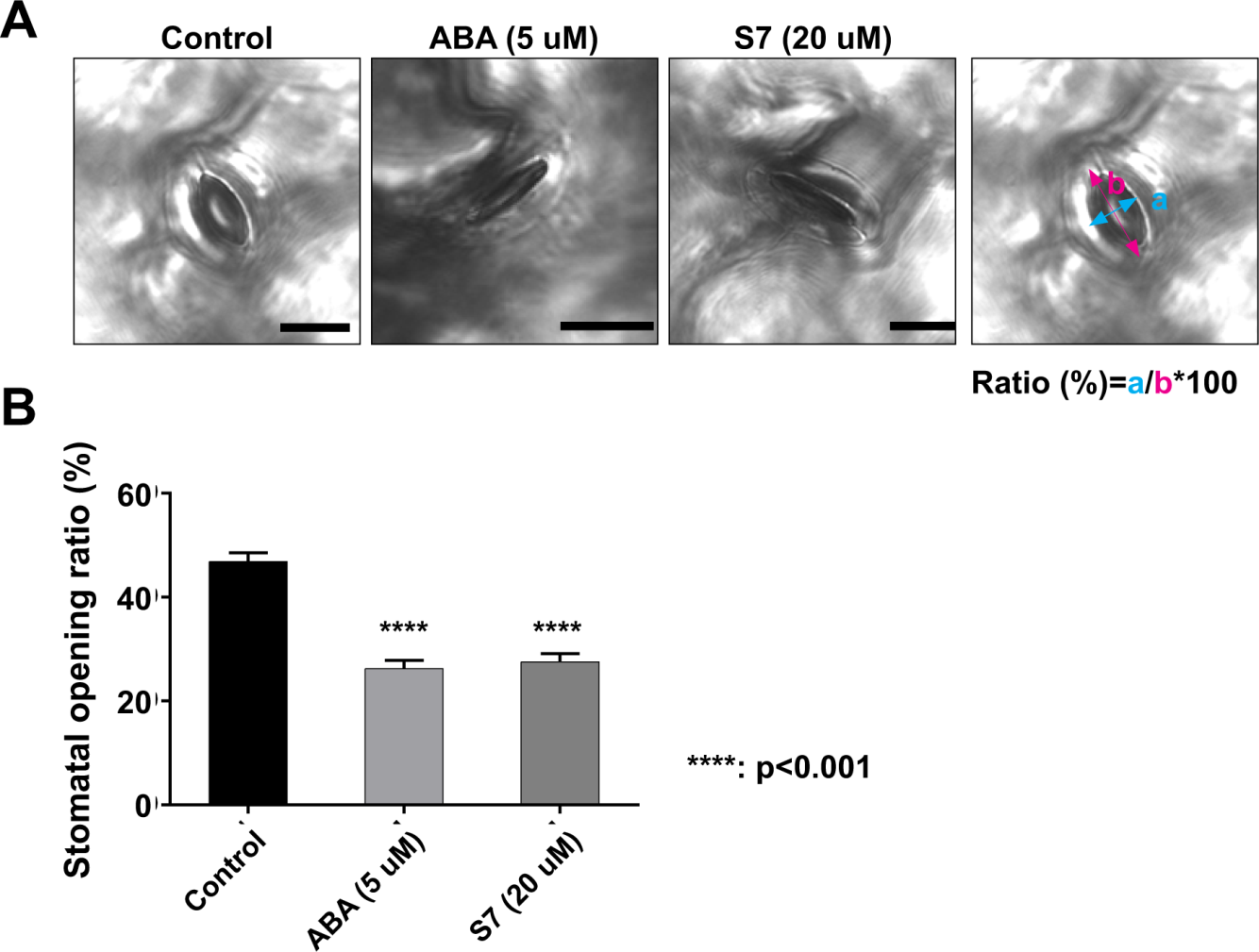
**

**Supplementary figure 2. Stomatal closing is induced by S7 or ABA**. (A) Confocal images of stomata with the treatment of indicated chemicals. 4 week-old Arabidopsis leaves were detached and incubated in D.W containing chemical for 3h under day light condition. The stomata images were captured using Leica TCS SP8 confocal microscopy. Size bars are 10 μm. (B) Stomatal opening ratio of chemical treated samples. At least 20 stomata were observed at each 3 times replication. The statics were analyzed with one way ANOVA for multiple comparing, ****=P<0.0001. Error bar= SE.


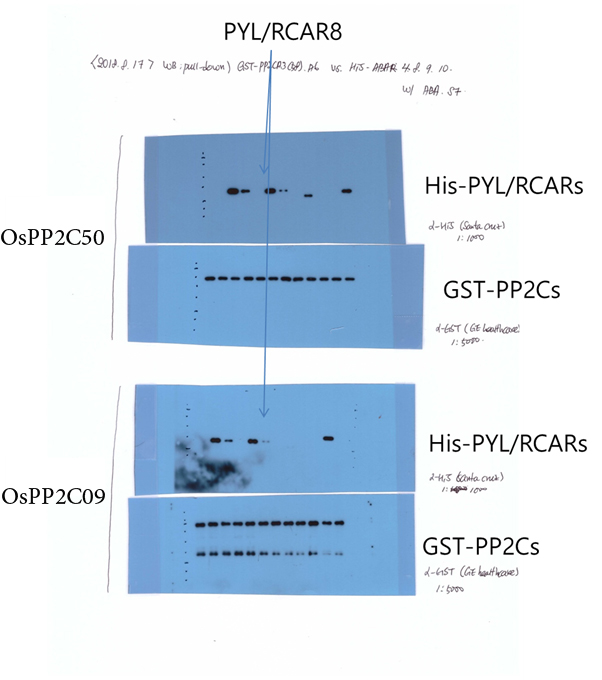


**Supplementary Figure 3. Pull-down assay of GST-tagged PP2Cs and His-tagged OsPYL/RCARs under S7 and ABA treatment.**

Pull-down assay of GST-tagged proteins using GSH-conjugated agarose beads. The proteins were detected using a western blot analysis with anti-His mouse antibodies and anti-GST rabbit antibodies
